# Special issue: e-health innovations for global mental health

**DOI:** 10.1017/gmh.2018.6

**Published:** 2018-02-01

**Authors:** T. Donker, A. Kleiboer

**Affiliations:** Department of Clinical, Neuro and Developmental Psychology, Amsterdam Public Health Research Institute, Vrije Universiteit Amsterdam, Amsterdam, The Netherlands

A staggering amount of 25% of people worldwide are estimated to be affected by mental in their lives (WHO, 2011). Although effective interventions to reduce mental health symptoms exist, many sufferers do not receive mental health care. In particular, as much as 76–85% of people in need of treatment in low- and middle-income countries (LMICs) do not receive any treatment at all (Patel *et al.* 2011; WHO, 2013). Estimations may even be worse; Patel *et al.* (2016) revealed a treatment gap exceeding 90% for common mental disorders and alcohol use disorders in India and China, two relatively well-resourced middle-income countries (Fairburn & Patel, 2016). The lack of a mental health policy, mental health programmes and mental health legislation in many countries, as well as limited resources (both financial and human), a limited infrastructure, stigma and shame are important reasons for low uptake and dissemination of mental health care (WHO, 2008; Munoz *et al.* 2016). While internet penetration and mobile phone ownership in particular is increasing globally (Pew Research Center, 2016), digital technologies offer a genuine opportunity to overcome several of these barriers.

e-Health technologies have been extensively researched in the last few decades. Numerous systematic reviews and meta-analysis of randomized controlled trials have demonstrated the effectiveness of guided and unguided e-health interventions for, e.g. depression, anxiety, alcohol disorders and insomnia (Cuijpers *et al.* 2010; Richards & Richardson, 2012; Andersson & Titov, 2014; Riper *et al.* 2014; Karyotaki *et al.* 2017). Unguided digital treatments, especially, have the potential to reach millions of underserved people because of its scalable nature. For example, the online depression intervention ‘MoodGYM’ has been used by over three-quarters of million people since 2001 (Christensen *et al*. 2002; Fairburn & Patel, 2016). Although completion rates are low and effects are small as there is no therapist support, the public health impact may still be large. Recently, online clinics such as MindSpot for Australians with depression and anxiety (Titov *et al.* 2015) have emerged. Such clinics are likely to proliferate as they have many advantages for both users (e.g. ease of access) and healthcare systems (e.g. high patient throughput at low cost) (Fairburn & Patel, 2016). Digital treatment provided through mobile apps, virtual reality, serious gaming and artificial intelligence will furthermore become readily available in the nearby future to treat mental health problems (Fairburn & Patel, 2016).

The adoption of e-health technologies has the potential to revolutionize the delivery of mental health education, training, care and advocacy in low resource settings. The goal of the current issue of Global Mental Health is to illuminate on current applications and promising new directions in e-mental health relevant to low resource settings. We present overview and review papers that summarize the research literature, as well as original research papers elaborating on recent innovations in the area. We therefore hope that this issue will contribute towards filling the knowledge gap of barriers of implementing effective treatment in LMICs as well as pointing out the needs and opportunities e-health technologies have to offer in LMICs.

In their overview of low-intensity and online interventions for depression in LMICs, first Bockting *et al.* (2016) propose three strategies for addressing the mental health gap in LMICs, which is the enormous disparity between the number of individuals in need of mental health care and the availability of professionals of such care. These are the delivery of evidence-based, low-intensity interventions by non-specialists, the use of transdiagnostic treatment protocols and strategic deployment of technology to facilitate access and uptake.

The paper by Ruzek & Yeager (2017) provides a critical review of the literature on online and mobile technology interventions of mental health for trauma survivors in less resourced communities. Evolving strategies for strengthening mental health response to the needs of trauma survivors in LMICs are explored, and key challenges related to implementation and policy are elaborated on.

In terms of feasibility, Sobowale *et al.* (2016) explored the perceptions of Vietnamese youth and parents towards digital interventions for youth mental health as a first step to implement internet-based treatment in Vietnam.

Finally, digital technologies may offer potentially new ways in obtaining more accurate reporting of sensitive behaviours such as substance use and sexual risk behaviours compared with interviewer-administrative questionnaires. Kane *et al.* (2016) describe their experience in implementing an audio computer-assisted self-interviewing system for use with a population of orphans and vulnerable children in Zambia. If feasible and effective, Audio Computer-Assisted Self-Interviewing (ACASI) may be an effective and economical alternative for behavioural health research studies in LMICs.

These papers contribute significantly in identifying knowledge gaps and aim to give direction for policy makers and mental health care workers to increase accessibility of evidence-based mental health care to those in need, as well as sharpening future directions in implementing e-health technologies in LMICs. However, although the papers highlight the potential of digital mental health care, more rigorous research is needed to examine the feasibility, effectiveness, and implementation and dissemination process of these interventions in LMICs if digital health interventions want to live up to their promise.
